# Investigation of ANN architecture for predicting the compressive strength of concrete containing GGBFS

**DOI:** 10.1371/journal.pone.0260847

**Published:** 2021-12-03

**Authors:** Van Quan Tran, Hai-Van Thi Mai, Thuy-Anh Nguyen, Hai-Bang Ly

**Affiliations:** University of Transport Technology, Hanoi, Vietnam; RMIT University, AUSTRALIA

## Abstract

An extensive simulation program is used in this study to discover the best ANN model for predicting the compressive strength of concrete containing Ground Granulated Blast Furnace Slag (GGBFS). To accomplish this purpose, an experimental database of 595 samples is compiled from the literature and utilized to find the best ANN architecture. The cement content, water content, coarse aggregate content, fine aggregate content, GGBFS content, carboxylic type hyper plasticizing content, superplasticizer content, and testing age are the eight inputs in this database. As a result, the optimal selection of the ANN design is carried out and evaluated using conventional statistical metrics. The results demonstrate that utilizing the best architecture [8–14–4–1] among the 240 investigated architectures, and the best ANN model, is a very efficient predictor of the compressive strength of concrete using GGBFS, with a maximum R^2^ value of 0.968 on the training part and 0.965 on the testing part. Furthermore, a sensitivity analysis is performed over 500 Monte Carlo simulations using the best ANN model to determine the reliability of ANN model in predicting the compressive strength of concrete. The findings of this research may make it easier and more efficient to apply the ANN model to many civil engineering challenges.

## 1. Introduction

Concrete is a type of building material that is extensively used worldwide thanks to its various advantages. Therefore, the investigation of concrete mechanical properties is very crucial in designing concrete structures. In which, compressive strength is the most important property because the compressive strength is directly influenced by the safety and performance of the structure during the whole life-cycle for both old and new structures [1]. Nonetheless, concrete is created by different components such as aggregates, cement, supplementary cementitious materials, additional mixtures, which are all randomly distributed in the concrete matrix. As a result of the complexity of concrete structure materials, precisely estimating the concrete compressive strength is extremely difficult [2].

Physical experiments are usually the most straightforward means of determining the concrete compressive strength. In most cases, cubic or cylinder specimens were made according to the mix design ratio and then cured for the specified amount of time. Then, the compressive test instrument is used for determining the compressive strength [3]. However, the experiment test is indeed time and money-consuming. As a result, construction efficiency will be severely impacted. For reducing the time-consuming and cost of experiment tests, some empirical models are proposed to predict the compressive strength of concrete with different components in concrete [4, 5]. However, the compressive strength and concrete components exhibit a strongly nonlinear relation. Therefore, an accurate regression expression is difficultly derived in predicting the concrete compressive strength. The different approach to estimate the concrete behavior is the numerical model [6, 7]. Li et al. [8] conclude that the reproduction of concrete behavior is complex and challenging due to the coupling of randomness and nonlinearity of each component and concrete compressive strength.

Machine learning (ML) algorithms have become prominent in various aspects of life in the last few decades, thanks to the rapid growth of artificial intelligence technology [9]. Among ML algorithms, ANN is a viable algorithm for resolving difficult technical problems at the moment [10, 11]. The ANN model can solve nonlinear and complex nonlinear problems. The link between the inputs and outputs, in particular, cannot be stated explicitly. The capacity of the ANN algorithm to self-learn and modify the weights is a significant benefit. As a consequence, without relying on mechanical equations, physical chemistry, or other factors, model findings are consistent. Many challenging civil engineering issues have been successfully solved, such as structure problems [12, 13], geotechnical [14–16], and materials [17–19]. Besides, Abdalla et al. [14] used an ANN model to effectively estimate the minimal safety factor against slope failure in clayey soils. The mechanical characteristics of FRP concrete may also be predicted with great accuracy using an ANN model [20, 21]. In materials science, ML approaches and ANN have been used to predict various concrete characteristics, such as concrete beams shear strength [22, 23], corrosion properties [24], crack [25], concrete beams ultimate strength [26], recycled aggregate concrete [27], silica fume concrete [28], concrete using blast furnace slag [29–34], or concrete using fly ash [35–38]. In the details, Bilim et al. [33] developed an ANN model to predict the compressive strength using 225 data points. In this study, R^2^ = 0.96 is the best value of prediction performance. Furthermore, Palika Chopra et al. [39] developed an ANN model with one hidden layer and 50 neurons to predict the concrete compressive strength utilizing 204 data points. The coefficient of determination R^2^ = 0.92 is used to measure the performance of such an ANN model. Yeh [40] developed an ANN model for forecasting the compressive strength of concrete using BFS and FA, with 1030 data points. The construction of the ANN network in the well-known contribution of Yeh consists of one hidden layer and eight neurons, and the prediction accuracy of such an ANN model is pretty high, with R^2^ = 0.922. Overall, the number of hidden layers and number of neurons in each hidden layer have a substantial impact on the performance of an ANN model [41]. The effectiveness of ANN model demonstrates that it is a good choice for developing a numerical tool for engineers to estimate the concrete compressive strength, potentially saving time cost and reducing experiment costs. As a result, the primary goal of this investigation is to develop an effective ANN model with an appropriate architecture for forecasting the concrete compressive strength with more accuracy.

The ANN model is utilized to predict the concrete compressive strength in this study. One of the most significant factors impacting the model’s performance is the ANN architecture. As a result, the major objective of this article is to examine and improve the ANN architecture for predicting the concrete compressive strength. The optimal ANN architecture is decided by the model’s performance, which is assessed using well-known statistical metrics, namely the coefficient of determination (R^2^), mean absolute error (MAE), root mean squared error (RMSE). Then, using Monte Carlo simulation (MSC), 500 runs are performed for each model, taking into consideration a random sampling effect to ensure that the suggested model is both feasible and convergent. In the final part, the features importance is also explored to illustrate the influence of each input variable on the compressive strength of concrete.

## 2. Database construction

**Fig 1:**
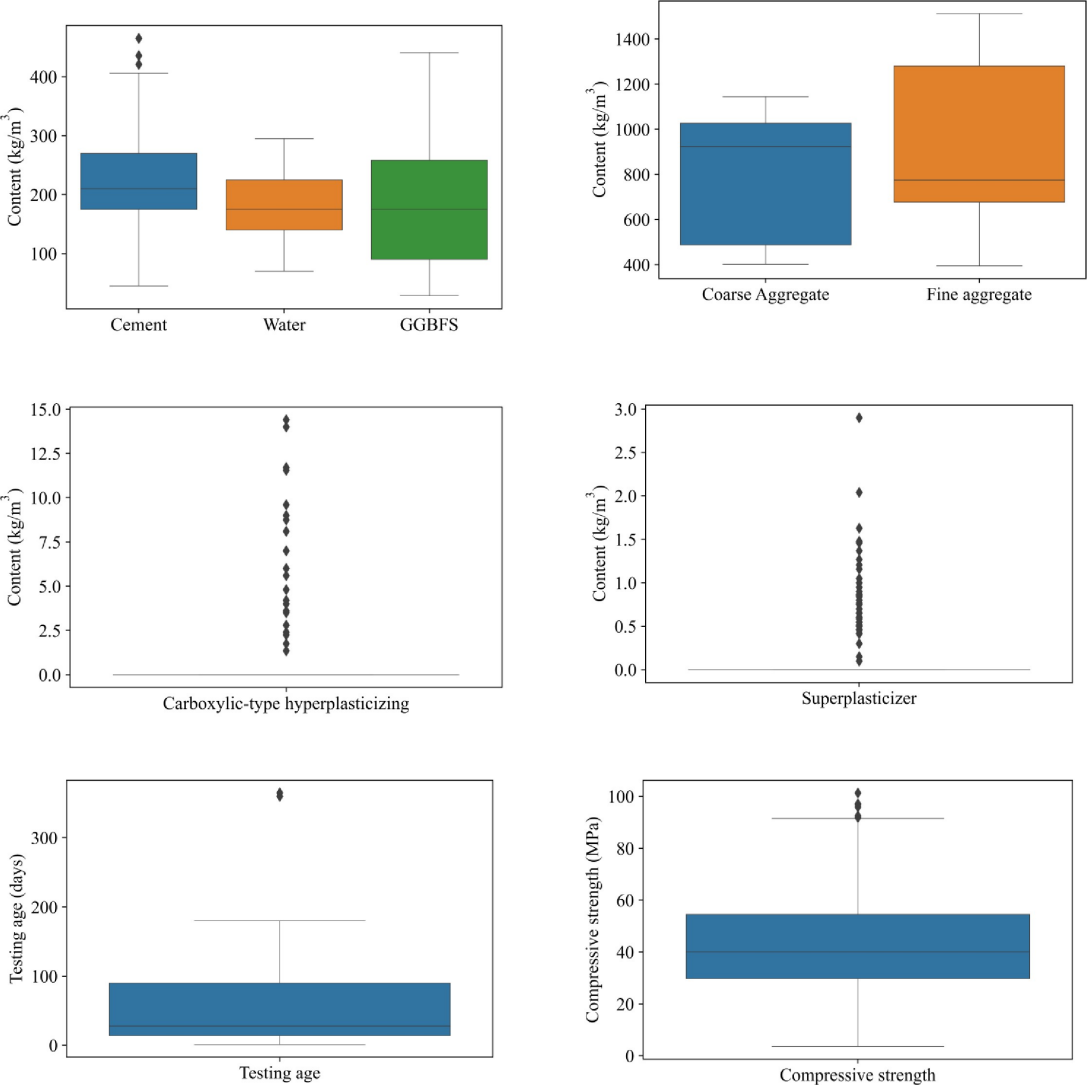
Boxplot describing input and output variable range.

**Fig 2:**
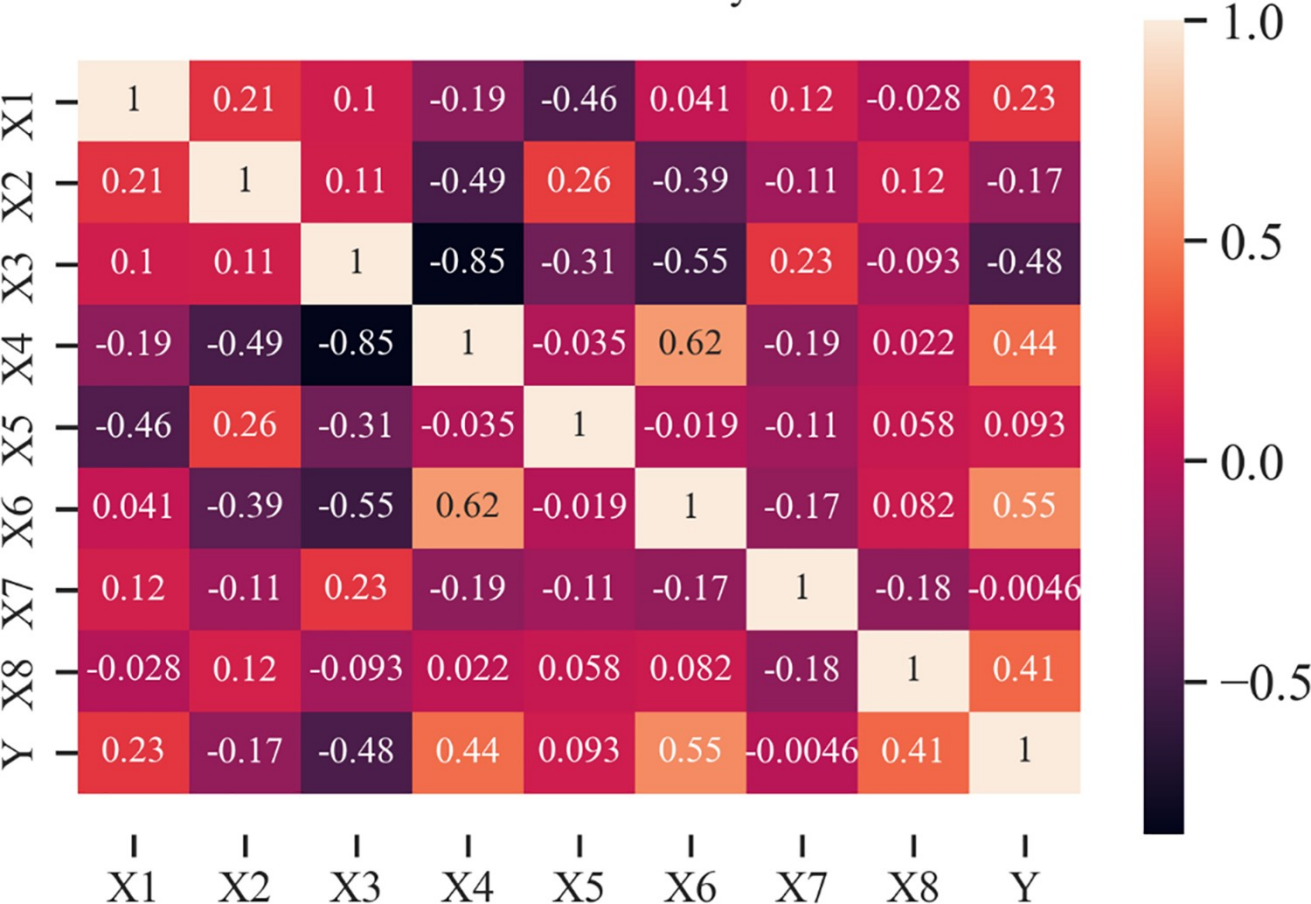
Correlation analysis of the input and output variables.

This study’s experimental database was collected from published papers [4, 32, 33, 42–44] (Table 1). There are 595 samples, divided into two parts, 70% training data corresponding to 417 samples and 30% testing data corresponding to 178 samples. There are two shapes of samples, including 36 cylindrical samples and 559 cubic samples, accounting for 93.9% of total samples. There are 8 input variables in the database, ranging from X_1_ to X_8_. They represent the binder content in the concrete mixture, such as cement (X_1_) or GGFBS (X_5_); the water content (X_2_); aggregate such as coarse (X_3_) or fine, sand contents (X_4_); admixtures contents such as carboxylic-type hyperplasticizing (X_6_) or superplasticizer (X_7_); and age of samples, expressed in day (X_8_). The considered output is the compressive strength, measured in MPa (denoted as Y). Fig 1 depicts the boxplots describing the range of each database input variable. The corresponding correlation analysis of data is shown in Fig 2.

**Table 1:** Detail of database collection.

No.	Reference	No. of data points	(%)
1	Oner and Akyuz [4]	168 samples in cubic form	28.22
2	Shariq et al. [42]	63 samples in cubic form	10.58
3	Chidiac and Panesar [43]	36 samples in cylindric form	6.10
4	Boga et al. [32]	6 samples in cubic form	1.00
5	Bilim et al. [33]	180 samples in cubic form	30.24
6	Han et al. [44]	142 samples in cubic form	23.86
**Total**		595 samples in cubic form	100

The input variables X_1_ to X_5_ have a broad range of values, whereas X_6_ to X_8_ have a narrow range of values. Cement content (X_1_) varies from 45 to 464 (kg/m^3^), with 218.352 (kg/m^3^) being the average. Water content (X_2_) varies between 70 and 295 kg/m^3^. As indicated in Table 1, coarse aggregate content (X_3_) ranges from around 402 to 1145 (kg/m^3^), with no sample falling between 500 and 700 (kg/m^3^). Fine aggregate content (X_4_) ranges from 395 to 1512 (kg/m^3^), with 680 kg/m^3^ being the highest frequented sample with such content. The composition of GGBFS (X_5_) ranges from 28 to 440 kg/m^3^. The carboxylic-type hyper-plasticizing content (X_6_) is measured in kg/m^3^ and varies from 0 to 14. Furthermore, with the exception of six samples (containing roughly 1% of the total), virtually all samples have no superplasticizer content (X_7_). There are ten options for the sample age (X_8_), the sample’s lowest age is one day, and the sample’s maximum age is 365 days. Table 2 provides more detailed information on these values and ranges.

**Table 2:** Summary of the input and output variables.

	Sym.	Unit	Min	Median	Mean	Max	StD^a^	SK^b^
Cement (X_1_)	X_1_	kg/m^3^	45.000	210.000	218.352	464.790	70.934	0.004
Water (X_2_)	X_2_	kg/m^3^	70.000	175.000	181.305	295.000	53.060	0.019
Coarse Aggregate (X_3_)	X_3_	kg/m^3^	402.270	923.000	820.902	1145.000	254.271	-0.494
Fine aggregate (X_4_)	X_4_	kg/m^3^	395.000	775.000	929.797	1512.675	324.483	0.484
GGBFS (X_5_)	X_5_	kg/m^3^	28.667	175.000	181.547	440.697	95.631	0.518
Carboxylic-type hyperplasticizing (X_6_)	X_6_	kg/m^3^	0.000	0.000	1.229	14.400	2.994	2.758
Superplasticizer (X_7_)	X_7_	kg/m^3^	0.000	0.000	0.158	2.900	0.389	3.445
Testing age (X_8_)	X_8_	day	1.000	28.000	76.518	365.000	106.088	1.913
Compressive strength (Y)	Y	MPa	3.590	40.100	43.298	101.300	19.024	0.599

^a^ = Standard deviation

^b^ = Skewness; Sym. = Symbol.

Fig 2 depicts various correlations between the inputs and output Y (compressive strength). The correlation values are given in different colors, depending on the associated values. Some of the variables, such as X_4_ and X_6_ for aggregate and carboxylic-type hyper plasticizing contents, are marginally correlated, as shown. Overall, however, the correlation between inputs and compressive strength is poor. As a result, all factors are taken into account in order to improve the suggested ANN model’s accuracy and generalization capability. It is worth noting that all of the samples in the present database have undergone a traditional curing procedure (i.e., stored in laboratory conditions up to the testing date). To guarantee data point consistency, any samples with unusual curing conditions are deleted from the database. If it is considered, another input variable must be added into the input space to represent the curing process, which may be the goal of future study. In this research, the sample size for performing compressive strength tests is ignored. Indeed, the database’s sample size is mostly in cubic shape, with just 36 samples examined with cylindrical ones. It has been shown previously [18] that the effect of sample size may be ignored when only cubic and cylindric samples are used to estimate compressive strength. In situations when there are several kinds of sample size, additional input parameter may be required to properly represent the prediction process.

## 3. Methods

### 3.1. Artificial neural network

**Fig 3:**
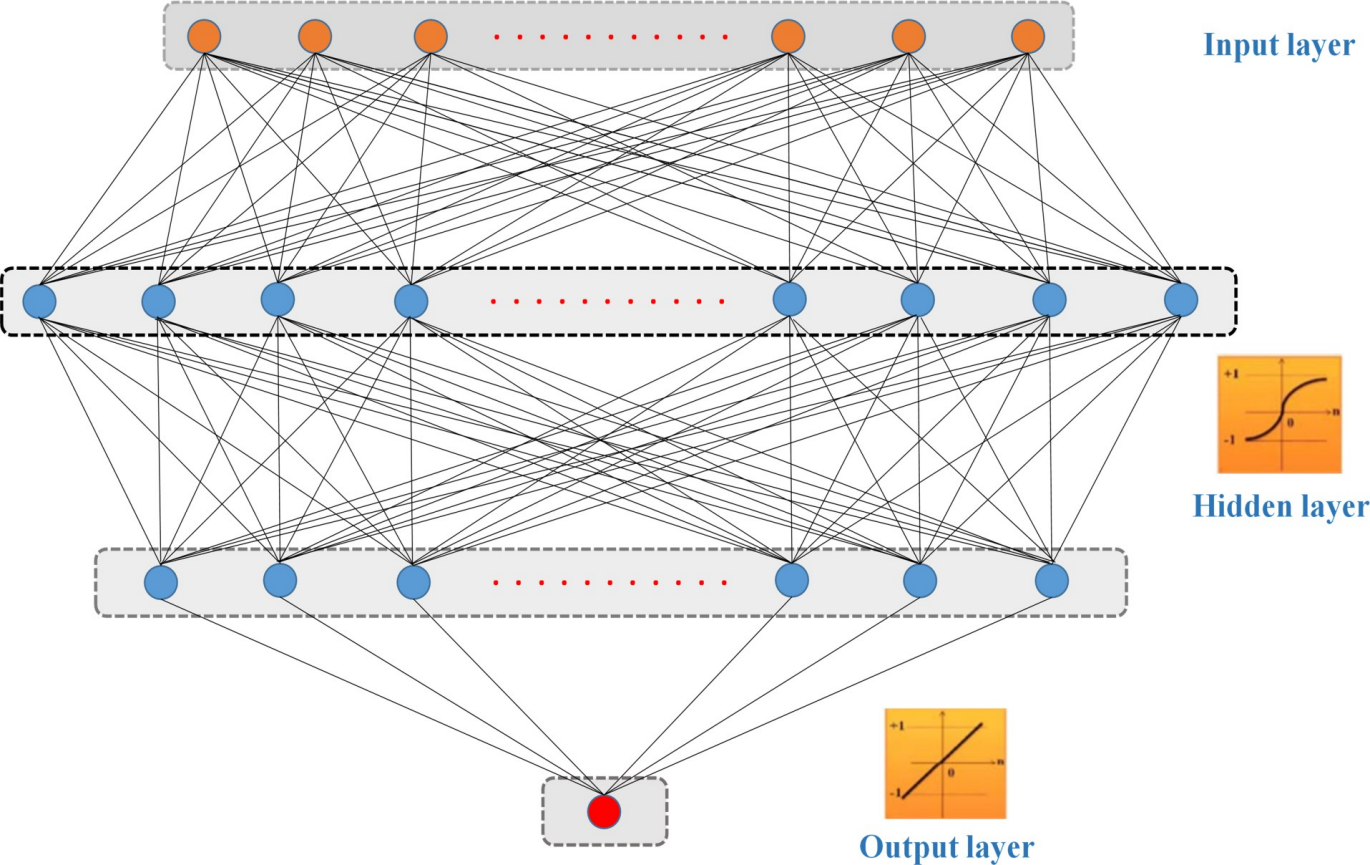
An ANN framework used in this research.

As the name implies, an artificial neural network (ANN) consists of a collection of data analysis techniques that enable the complicated mathematical connection between a collection of influencing factors (Inputs) and a variable or a collection of target variables to be discovered. Because of ANN’s structure and learning, inference, and regeneration processes, it can dynamically adapt to a wide variety of data sources. ANN uses the information processing technique of biological neuron networks in the human brain to process it. To process and evaluate information, it is made up of numerous neurons linked by weighted connections. Using a collection of training patterns, an artificial neural network is built for a particular purpose (pattern recognition, data categorization, regression). The input data will be analyzed by the whole neural network, relationships will be found, and the output will be reconstructed. These results will be compared to what the system has already learned about the target data set in the past. This training-learning procedure will be repeated if there is still a substantial difference in output between what was expected (the target) and what was actually obtained (the deviation from the target). This cycle is performed as many times as necessary to get the lowest feasible deviation between the output and the target. Hsu et al. stated that [45], with its versatile mathematical function structure, the ANN network is an excellent estimator. Therefore, a correlation between input and output may be shown in any system using this technique. Fig 3 depicts the basic ANN structure, which includes three or more layers of neurons.

Backpropagation learning was utilized by a number of methods to train the ANN model, such as gradient descent [46], Levenberg-Marquardt [47], and Scaled Conjugate Gradient (SCG) [48]. The method through which the weights and biases of the network are adjusted differs across the training methods [49]. According to Moller [48], in comparison to other algorithms, the SCG method achieves a quicker convergence speed because it employs the suitable extreme detection step ratio mechanism. Using the scaled conjugate gradient technique, the SCG algorithm is used as a network training function, updating the weight and bias values of the network. Any network having derivative functions in its weights, network inputs, and transfer functions may be trained using SCG. Consequently, the SCG method is used as the ANN model’s training function in this research.

### 3.2. Performance criteria

Different statistical metrics, such as the coefficient of determination (R^2^), Mean Absolute Error (MAE), and Root Mean Squared Error (RMSE), are utilized to confirm and assess the performance of the ANN-SCG model. In regression issues, the R^2^ criterion is commonly employed to measure the correlation between the target and expected outputs [50]. Furthermore, MAE and RMSE are used to assess model error [51, 52]. In general, higher R^2^ illustrates the better predictive capability of the model, whereas lower RMSE and MAE show represent the higher accuracy of the model [53, 54]. Calculation of R^2^, RMSE, and MAE is based on the following equations:

$$R^{2}=\frac{\sum_{k=1}^{M}\left(V_{0,k}-{\bar{V}}_{0})(V_{t,k}-{\bar{V}}_{t}\right)}{\sqrt{\sum_{k=1}^{M}{\left(V_{0,k}-{\bar{V}}_{0}\right)}^{2}}\sum_{k=1}^{M}{\left(V_{t,k}-{\bar{V}}_{t}\right)}^{2}}$$ (1)

$$MAE=\frac{\sum_{k=1}^{M}\left|V_{0,k}-V_{t,k}\right|}{M},$$ (2)

$$RMSE=\sqrt{\frac{1}{M}\sum_{k=1}^{M}{\left(V_{0,k}-V_{t,k}\right)}^{2}}$$ (3)

where *M* is the number of the samples, *V*_*0*,_ and ${\bar{V}}_{0}$ is the actual value and the average experimental value, *V*_*t*_ and ${\bar{V}}_{t}$ is the predicted value and the average predicted value determined using the prediction model (k = 1:M). (*k* = 1:*M*).

## 4. Methodology flow chart

**Fig 4:**
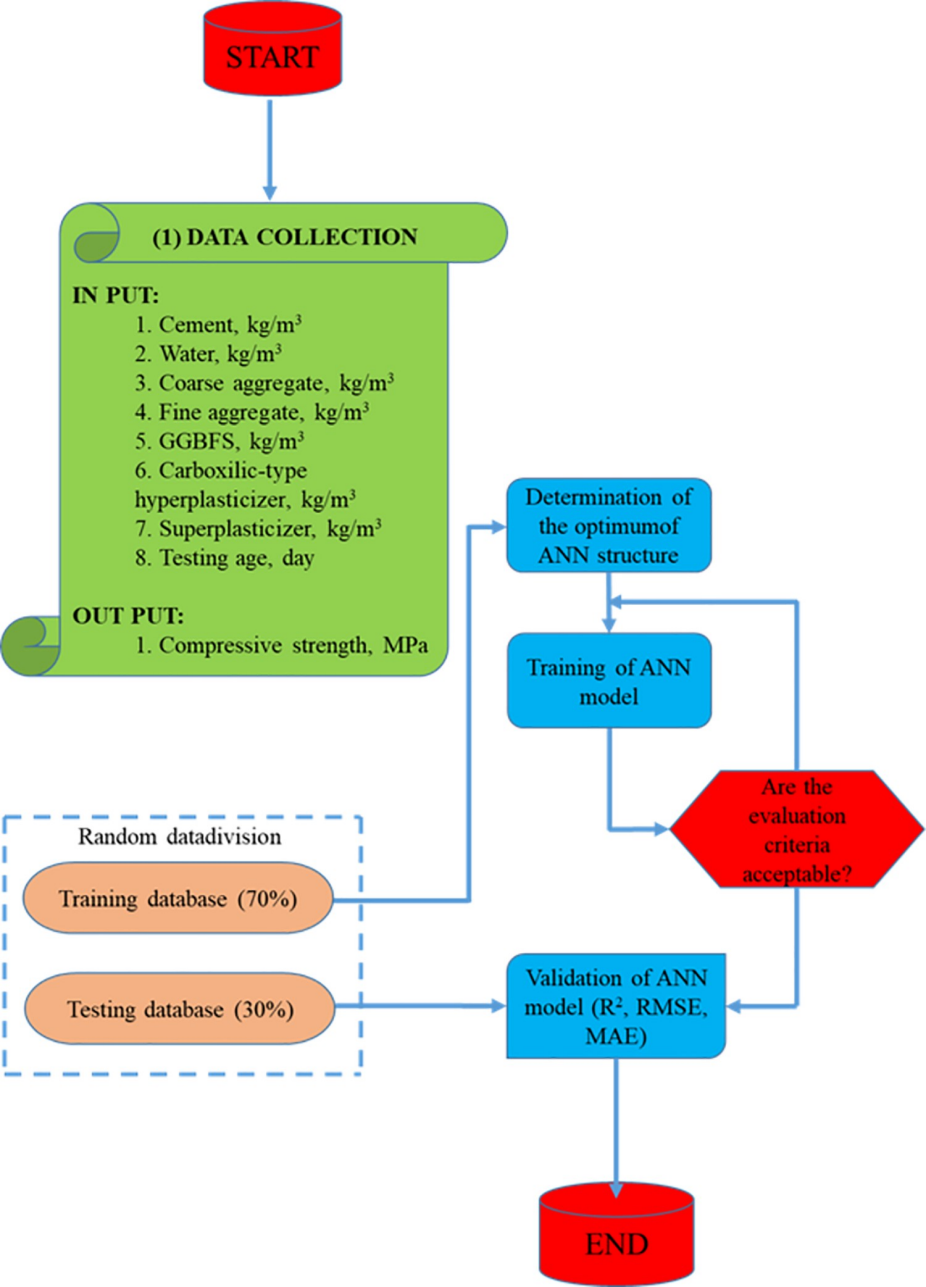
Methodology flow chart.

As illustrated in Fig 4, the process for constructing the ANN-SCG model to predict the compressive strength of concrete containing GGBFS consists of four steps:

1st step: The database collecting task is the initial phase. The ANN model is developed using a database of 595 samples. The data set is divided at random into two parts: 70% of the total data is used to train the ANN model, and 30% of the remaining data is used to test the ANN model.

Step 2: Determining the best ANN architecture. The creation of the best ANN structure based on the training data set is carried out in this second stage.

Step 3: ANN model training. The ANN model with the best architecture is trained using the training dataset and the Conjugate Gradient technique in the third stage.

Step 4: ANN model validation. The testing dataset is utilized in this final phase to verify and confirm the trained ANN model. Three statistical measures, R^2^, RMSE, and MAE, are used to assess the performance of the ANN model.

## 5. Results & discussion

The performance of the ANN model is determined by the structure of the neural network (NN), with the number of hidden layers and the number of neurons in each hidden layer being two important criteria. Typically, the number of hidden layers is defined initially during the ANN network structure design, and then the number of neurons in each hidden layer is chosen based on the complexity of the relationship between input parameters and output values. Unfortunately, there is no common rule for determining the number of hidden layers and the number of neurons inside each hidden layer. As a result, networking based on trial-and-error experiments is required to determine the ideal network setup.

In this investigation, the number of hidden layers varies from 1 to 2, and the number of neurons in each hidden layer changes from 1 to 15. In fact, numerous authors proposed different formulas to estimate number of neurons in a single hidden layer, such as Paola (1994) [55], Ripley (1993) [56], Sheela (2013) [41], Nagendra (1998) [57], Wang (1994) [58], Popovics (1990) [59], Neville (1986) [60]. Based on the number of input and output variables, the highest number of neurons is equal to 9 according to the proposed formula of Nagendra [57] (number of neurons = number of input + number of output). In the case of two hidden layers, the number of neurons is chosen based on the number of neurons in the case of single hidden layers to easily compare the effectiveness of hidden layer numbers. Therefore, the number of neurons in two cases is proposed in the range between 1 to 15 to cover the number of neurons suggested in the literature and minimize the computation time.

Network training is conducted for each network structure. An epoch is a single cycle of propagating all training patterns across a backpropagation network. The training procedure is continued until the network output error reaches an acceptable level (less than the initial specified error threshold). The goal of this method is to reduce the difference between the experimental data and the model output data. The trial-and-error method is also used to establish the ideal number of epochs for the ANN-SCG model. As a consequence, the optimization procedure is repeated 1000 times to tune the neurons’ weights and biases for each ANN structure. Besides, each ANN structure is performed with 500 different simulations in changing the sample index in the training data set to obtain reliable results. This means that with the existing data set, 417 samples will be randomly taken to build the training data set, and the remaining 178 samples will be used to test the predictive capability of the proposed ANN-SCG model. A total of 120,000 simulations are performed for 240 architectures, corresponding to 15 structures with one hidden layer and 225 structures with two hidden layers. The activation function in the hidden layer is the sigmoid function, and for the output layer is a linear function. The parameters of the ANN-SCG model used in this study are detailed in Table 3.

**Table 3:** Summary of different ANN characteristics and investigation parameters in this study.

Parameter	Parameter	Description
Fix	Input layer neurons	8
	Neurons in the output layer	1
	Activation function for hidden layers	Sigmoid
	Activation function for the output layer	Linear
	Cost function	Mean Square Error (MSE)
	Number of epochs	1000
	Number of simulations	500
	Training algorithm	Scaled conjugate gradient backpropagation (SCG)
Parametric study	Number of hidden layers	1 and 2 hidden layers
	Neurons in hidden layer	From 1 to 15 neurons in each hidden layer

### 5.1. Prediction performance of different ANN architectures

**Fig 5:**
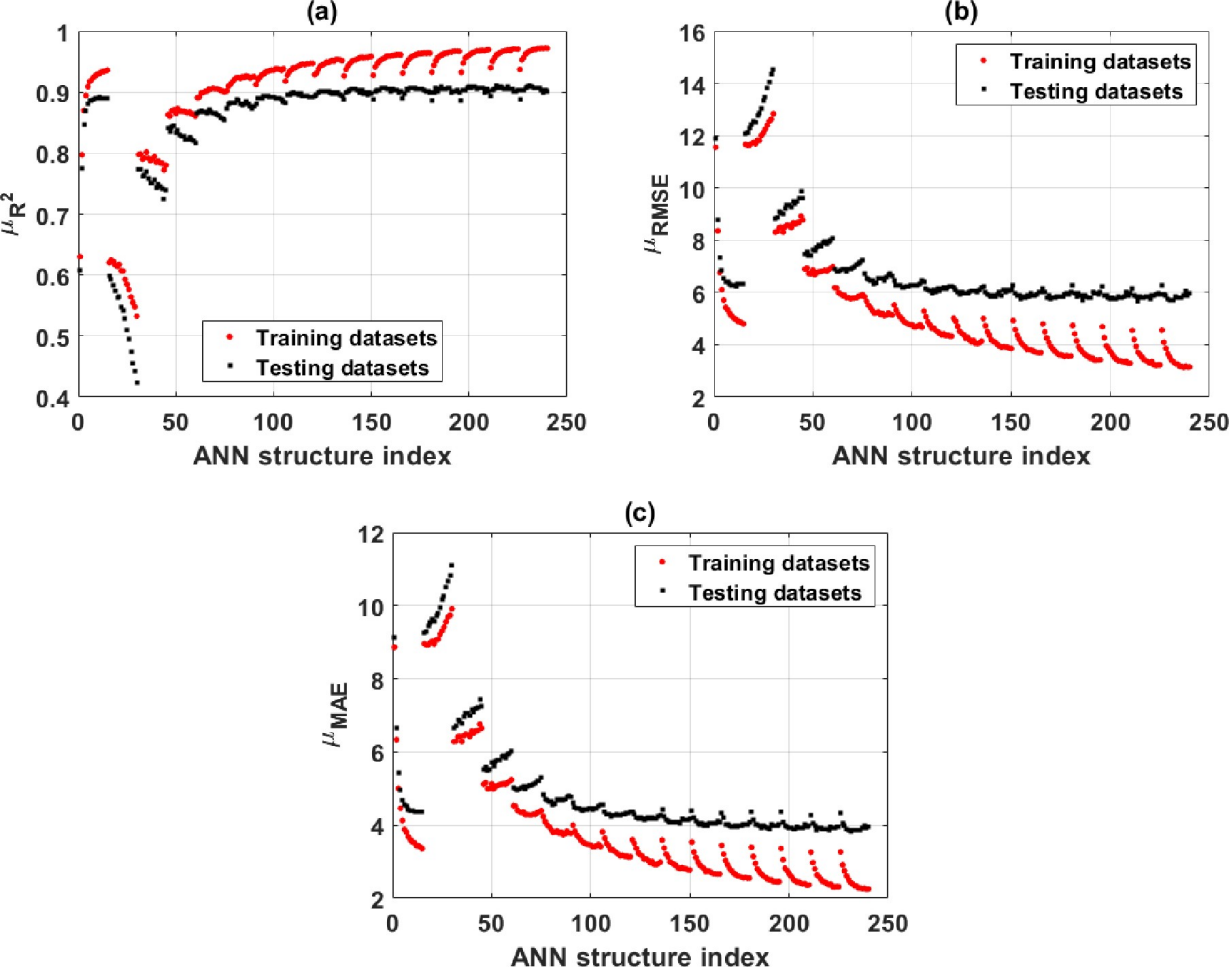
Performance of the ANN as a function of neuron count in two hidden layers, as measured by (a) mean R^2^ for the training and testing parts; (b) mean RMSE for the training and testing parts; and (c) mean MAE for the training and testing parts.

For the purpose of pinpointing the optimal ANN structure, it is necessary to compare the performance of several ANN architectures. R^2^, RMSE, and MAE metrics are used to evaluate the overall performance of the structures in question. In this section, 240 ANN designs are compared to see which one performs the best. The mean values of R^2^, RMSE, and MAE are used to evaluate performance in the training and testing phases. All ANN designs’ mean values for R^2^, RMSE, and MAE are shown in Fig 5A, 5B and 5C, respectively, for training and testing datasets.

The first edge of the curve in Fig 5 corresponds to the mean values of R^2^, RMSE, and MAE, representing an ANN model’s performance with one hidden layer and a neuron count ranging from one to fifteen. The second edge denotes the two-layer ANN architecture. The first point on the second edge is the performance of an ANN model with one neuron in the first hidden layer and one neuron in the second hidden layer, or an ANN architecture [8–1–1–1]. Each point along the remaining edges represents the performance of an ANN designed with one neuron in the first hidden layer, and the following points represent the performance of an ANN architecture with one to fifteen neurons in the second hidden layer. That is, the second edge’s final point corresponds to the ANN structure’s performance [8–1–15–1]. In general, one edge is displayed for an ANN designed with one hidden layer and fifteen edges for an ANN architecture with two hidden layers (Fig 5).

**Fig 6:**
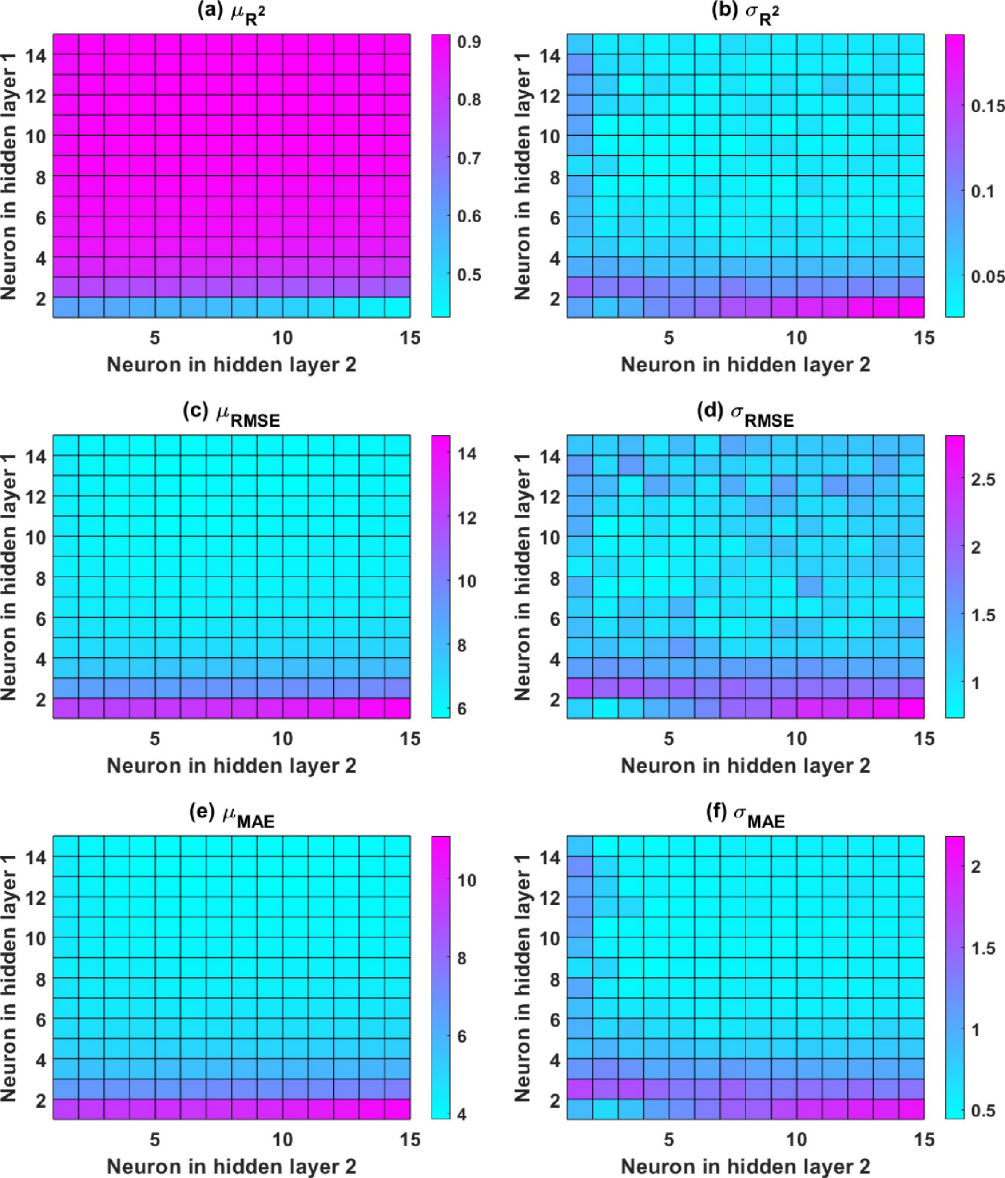
Color-map of ANN with two hidden layers for the testing part in relation to (a) mean R^2^; (b) StD R^2^; (c) mean RMSE; (d) StD RMSE; (e) mean MAE; and (f) StD MAE.

As shown in Fig 5, the optimal performance of the case 1 hidden layer corresponds to an ANN design with a single hidden layer comprising 15 neurons. For the testing section of this ANN design [8–15–1], the mean values of R^2^, RMSE, and MAE are 0.89, 15.5, and 11.5, respectively. With two hidden layers, the performance of the ANN model improves somewhat as the number of neurons grows. However, once the number of neurons in the first hidden layer reaches 6, the performance of the ANN model seems to remain constant regardless of the number of neurons in the second hidden layer (Fig 6). The next paragraph provides a more thorough explanation. The scenario with two hidden layers performs optimally when R^2^, RMSE, and MAE are 0.91, 6.0, and 4.0, respectively. These mean values are clearly superior to those obtained using an ANN with a single hidden layer.

For a better description of ANN model performance in the case of 2 hidden layers, the performance values of 225 ANN architectures are described by color map in Fig 6 for the testing part. Only the results from the testing dataset are considered here because the testing data reflects the model’s accuracy and predictability in the regression problem. Fig 6A, 6C and 6E show the mean values (denoted as μ) of R^2^, RMSE, and MAE, respectively. For ANN structures with more than 4 neurons in the first hidden layer, the value of R^2^ is considerably higher than 0.9 for the testing dataset. Similar findings are made for a particular zone with low RMSE and MAE values for the testing components. When the number of neurons in the first hidden layer is equal to or higher than 6, the color values of R^2^, RMSE, and MAE seem to be more stable. The optimum area is seen when the number of neurons in the first hidden layer is between 10 and 15, and the number of neurons in the second hidden layer is higher than 3. In addition, the impact of the number of neurons in the two hidden layers is assessed using three statistical criteria’s standard deviation values. Fig 6B and 6D, and 6f represent the standard deviation values (denoted as σ) of the three criteria, respectively. The standard deviation value is the minimum for simultaneously all statistical criteria, respectively, when the number of neurons in the first layer is 14, and the number of neurons in the second layer is 4. It can be inferred that the optimal structure of the ANN-SCG model with two hidden layers has the form of [8–14–4–1]. Then, for the training dataset, the average values of R^2^, RMSE, and MAE are 0.961, 3.706, and 2.640, respectively. These values are 0.910, 5.665, and 3.870 for the testing dataset, respectively. These values are superior than those obtained using an ANN design [8–15–1]. As a result, in the next part, this architecture is utilized to estimate the compressive strength of concrete.

**Fig 7:**
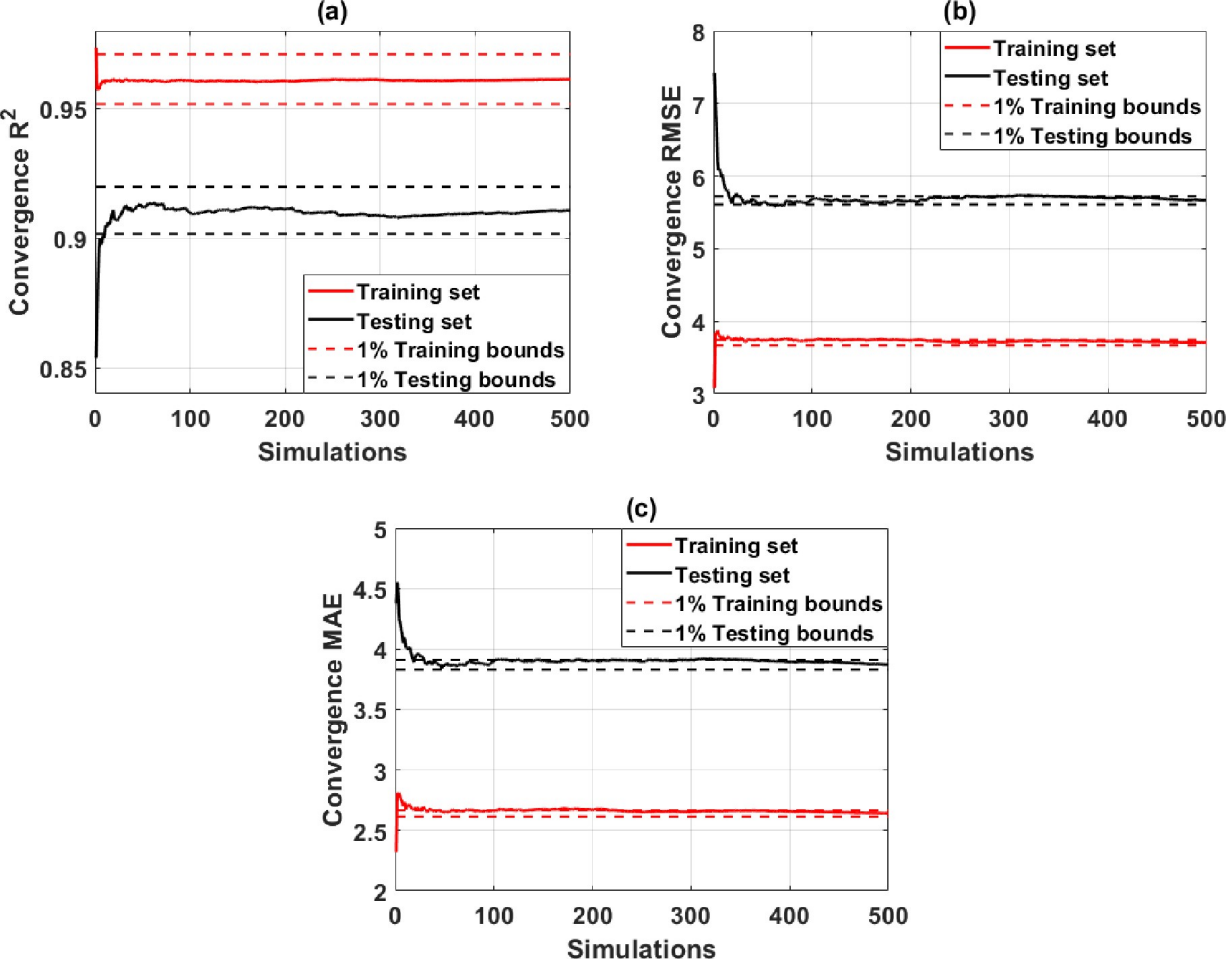
Convergence study of ANN [8–14–4–1] architecture in terms of (a) the R^2^ of the training and testing parts; (b) RMSE of the training and testing parts; (c) MAE of the training and testing parts.

Model ANN-SCG with structure [8–14–4–1] has been proven the best-structured model to predict the compressive strength of concrete, as mentioned above. The assessment of the convergence of simulations of the ANN-SCG model with the optimal structure is shown in Fig 7. The red line represents the average value of the statistical criteria for the training set, the black line for the testing dataset. The dashed line represents a 1% deviation around the mean value of the statistical criteria. As observed in Fig 7, after 30 simulations, the criteria achieved convergence within 1% around the convergence values. However, criterion R^2^ requires at least 350 simulations for the testing dataset to achieve convergence with small errors. With the RMSE criterion, a minimum of 300 simulations are required for the training dataset, and the MAE criterion requires a minimum of 200 simulations for the training and testing dataset. These analyses prove that with 500 simulations, under the random sampling effect of data is enough for the converged results obtained from the optimal ANN-SCG model.

### 5.2. Prediction performance of typical ANN architecture

**Fig 8:**
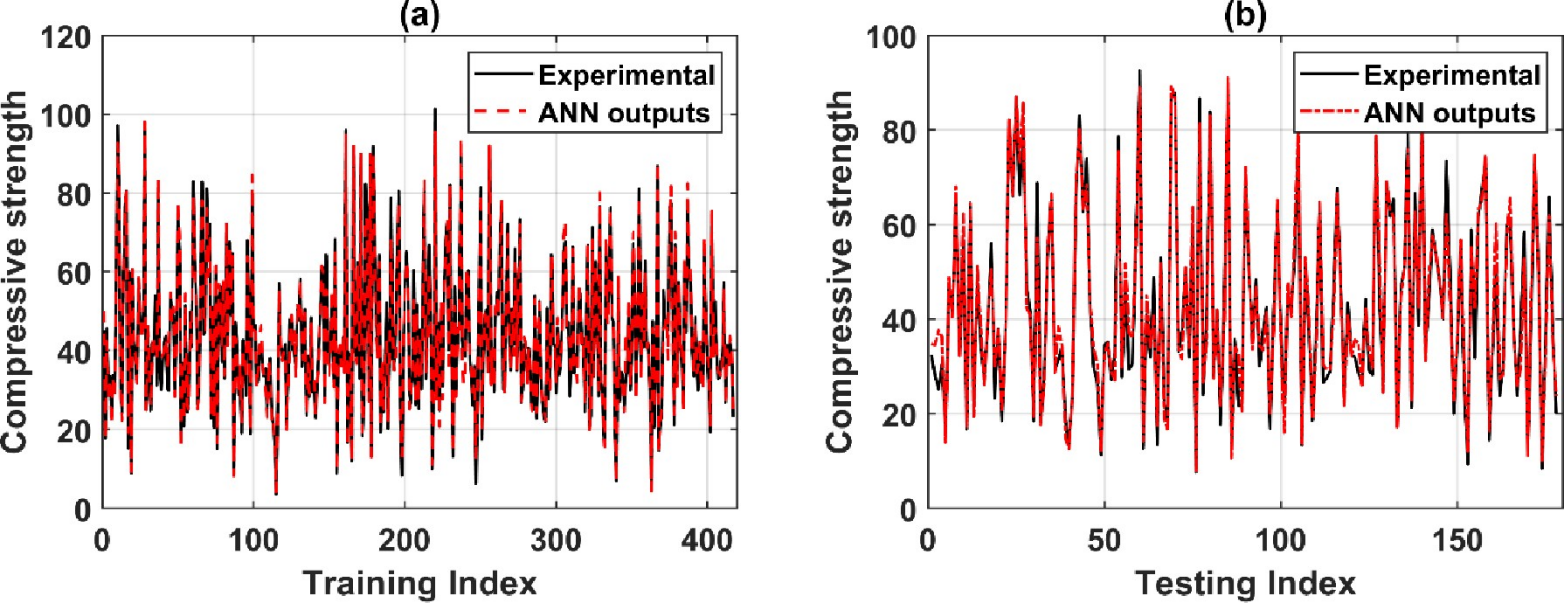
Experimental and predicted shear strength results in function of sample index for the training and testing datasets.

This section depicts a typical simulation to demonstrate the performance of the ANN-SCG model with the best architecture [8–14–4–1]. For training and testing dataset, the prediction results with the highest predictive capacity over 500 runs are shown. The relationship between concrete’s experimental compressive strength of concrete (red dashed line) and the predicted value (solid black line) from the training and testing parts is shown in Fig 8. In this figure, the horizontal axis indicates the number of samples in the data set, and the vertical axis denotes the compressive strength of concrete (MPa). The compressive strength of 417 samples in the training dataset is quite close to the actual results (Fig 8A). Regarding the testing dataset, 178 experimental results are also predicted with minor errors (Fig 8B). This accuracy is precisely quantified through the error values and the correlation between the experimental and the predicted results of the ANN-SCG model presented in the next section.

**Fig 9:**
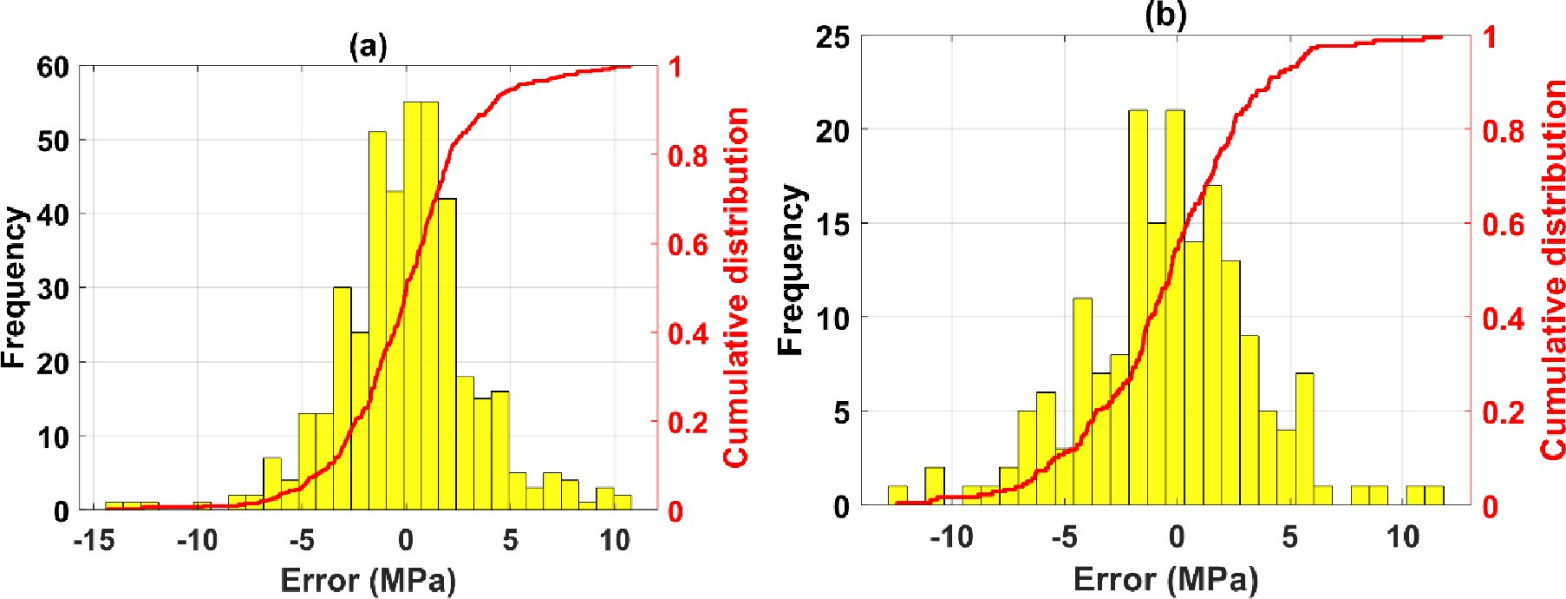
Experimental and predicted shear strength results in the function of sample index for the training and testing datasets.

The distribution and cumulative distribution of the error obtained by the ANN-SCG model [8–14–4–1] for the training set is shown in Fig 9A, and for the testing set is shown in Fig 9B. The error values between the training data and the experimental ones are small. Most of the error values are in the range [-5; 5] MPa, with very few samples having an error outside this range. Moreover, only 5 samples had an error outside the range [-10; 10] MPa, for both training and testing datasets. Based on the cumulative distribution (red line), it is easy to determine the samples’ percentage error within a range. For example, with the training dataset, the percentage of sample with the error between the experimental values and simulated ANN is in the range [-10; 10] MPa is 97%. This is similar to the testing dataset.

**Fig 10:**
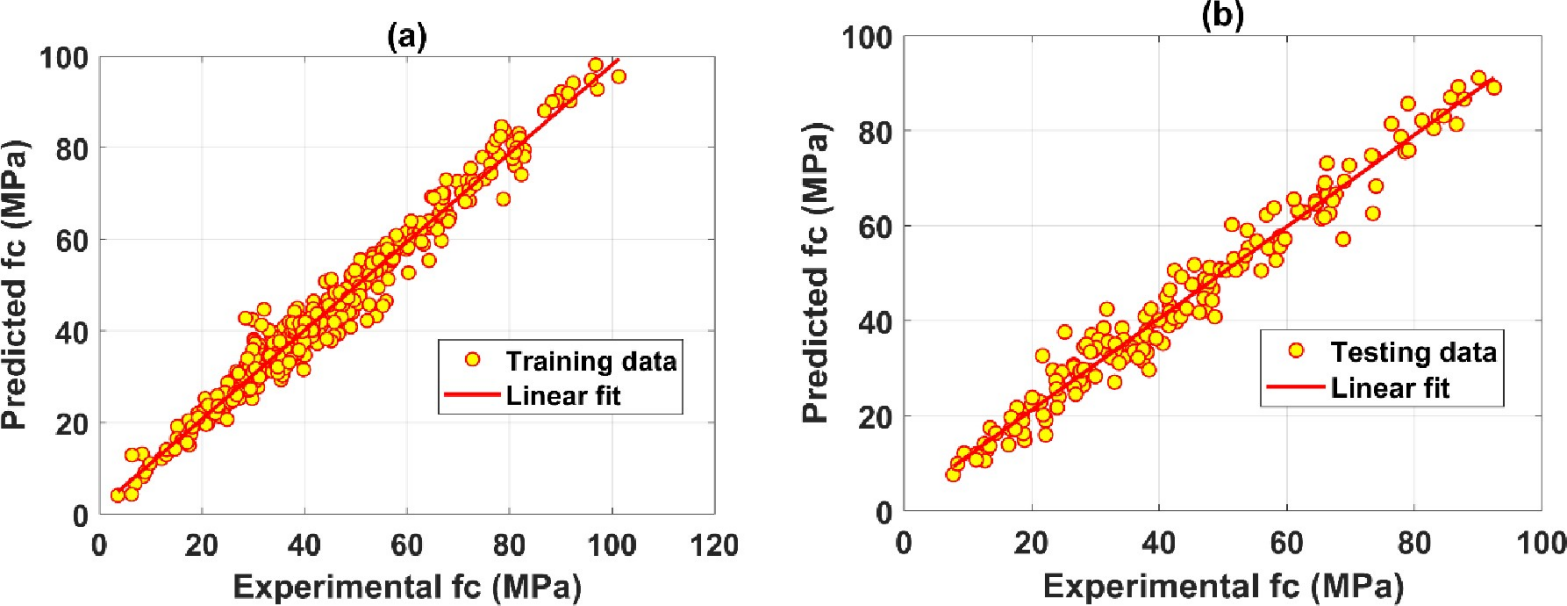
Regression graphs for the case of the best predictor ANN-[9–17–1]: (a) training dataset; (b) testing dataset.

Fig 10 shows the correlation analysis between the predicted value by ANN-SCG model and the experimental compressive strength value for the training and the testing datasets. As observed, the value obtained from the proposed model for the training dataset (Fig 10A) and the testing dataset (Fig 10B) is very close to the experimental results. These results show that the ANN-SCG model can successfully construct a relationship between input and output parameters and give good prediction results. Besides, the values of the three criteria for the training and testing data are presented in Table 4. The RMSE value is 3.284 and 3.803, respectively, for the training and testing dataset. The MAE value for the training set is 2.409, and the testing set is 2.880. The value of R^2^ is 0.968 corresponds to the training set, and the testing set R^2^ is 0.965. These values show that it is feasible to apply the ANN-SCG model to forecast the compressive strength of concrete containing GGBFS, saving time and costly experiments.

**Table 4:** Values of the best performance evaluation criteria of ANN-SCG model [8–14–4–1] for training and testing dataset.

	RMSE	MAE	R^2^
**Training dataset**	3.284	2.409	0.968
**Testing dataset**	3.803	2.880	0.965

For comparison purposes, Table 5 shows the prediction results in this work, with 6 results available in the literature. Saridemir et al. [34] used 5 inputs with 284 samples; therefore, the performance of the ANN model proposed is excellent with R^2^ = 0.980. Similarly, the investigation of Boğa et al. [32] could predict the compressive strength of concrete with a high value of R^2^ = 0.971 by ANN model using 162 samples and 4 inputs. It is worth noting that among the 6 compared investigations, these two models have higher R^2^ value than that of the ANN model proposed in this investigation. Besides, the RMSE value of ANN model proposed by Saridemir et al. [34] is lower than that in this investigation. However, the number of samples using in the ANN models proposed by Saridemir et al. [34] and Boğa et al. [32] are two times and 3.6 times less than the number of samples in the present work, respectively. Compared with Bilim et al. [33] and Han et al. [61], the authors used two times fewer samples than that this work, but the R^2^ values of the ML models are lower. The RMSE and MAE values in this investigation are higher than those proposed by Han et al. [61], but the difference is not significant. No comparison could be conducted with Bilim et al. [33] because the RMSE and MAE values are not published. Boukhatem et al. [30] and Kandiri et al. [31] collected 726 and 624 samples, respectively, for the development of some ANN models. The number of samples is slightly higher than this study (i.e., 595 samples), but the proposed ANN-SCG model shows higher performance R^2^ = 0.9650.

**Table 5:** Comparison of different machine learning models for predicting compressive strength of concrete containing GGBFS.

Reference	Machine learning algorithm	Input	No. of data	Performance measure
Saridemir et al. [34]	ANN model	5 inputs: TA, C, GGBFS, W and Agg.	284	R^2^ = 0.981
				RMSE = 2.511
Bilim et al. [33]	ANN model	6 inputs: C, GGBFS, W, SP, Agg. and TA	225	R^2^ = 0.96 (RMSE, MAE not available)
Kandiri et al. [31]	ANN and a multi-objective slap swarm algorithm (MOSSA)	7 inputs: C, GGBFS, W, fine Agg., coarse Agg., TA	624	R^2^ = 0.9409
				RMSE = 2.39
				MAE = 1.89
Han et al. [61]	ANN-PSO model	7 inputs: curing temperature, W/binder, GGBFS/total binder, W, fine Agg., coarse Agg., SP	269	R^2^ = 0.961
				RMSE = 3.332
				MAE = 2.689
Boukhatem et al. [30]	ANN model	5 inputs: C, W/C, GGBFS, temperature, TA	726	R^2^ = 0.9216 (RMSE, MAE not available)
Boğa et al. [32]	ANN model	4 inputs: cure type, curing period, BFS ratio, CNI ratio	162	ANN: R^2^ = 0.9710 (RMSE, MAE not available)
This work	ANN-SCG	8 inputs: C, W, coarse Agg, fine Agg, GGBFS, CH, SP, TA	595	R^2^ = 0.9650
				RMSE = 3.803
				MAE = 2.880

The accuracy of a machine learning model depends on numerous factors such as data distribution, sample size, training model and algorithm, optimization algorithm, number of input variables. In comparing the R^2^ value, the ANN model in the present study has higher R^2^ value than Boukhatem et al. [30]. The number of input variables of two models is different, with 5 inputs in Boukhatem et al. [30]. Furthermore, Boukhatem et al. [33] have considered the curing temperature, but not the main objective of the present work.

In comparing the model performance with Kandiri et al. [31], the R^2^ value of the ANN model proposed herein is higher than Kandiri et al. [31]. Moreover, the RMSE and MAE values are also lower than those obtained by the ANN model proposed by Kandiri et al. [31]. These comparisons confirm the feasibility and high accuracy of the ANN-SCG model proposed in this study. The comparisons (Table 5) could help material engineers in selecting the appropriate ANN model, depending on the input variable, to predict the compressive strength of concrete containing GGBFS.

### 5.3. Sensitivity analysis

**Fig 11:**
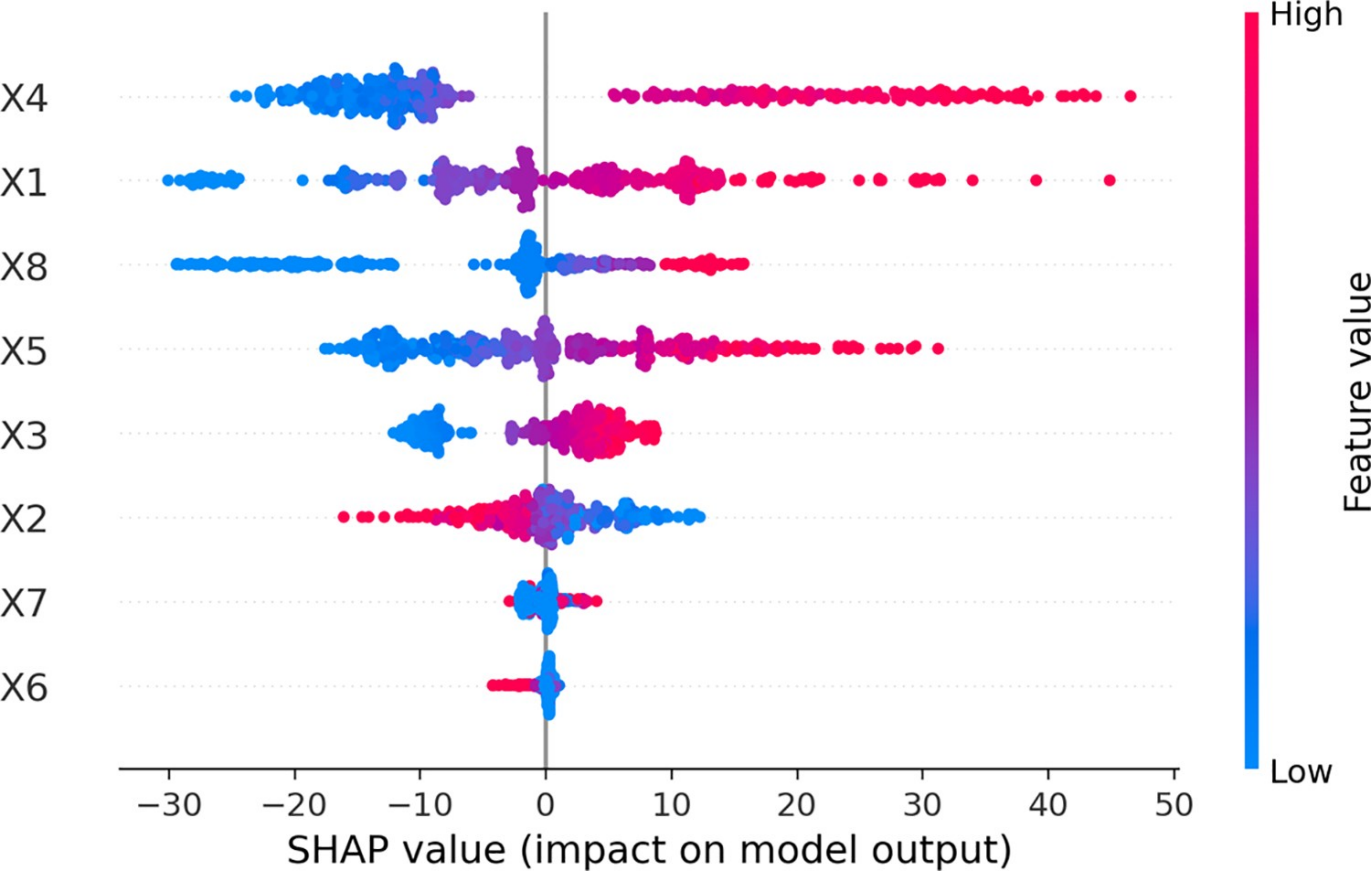
Feature importance of 8 variables used in this investigation.

The ANN model can evaluate the importance and effect of the input variable on the model’s prediction accuracy. The SHAP values are used to simulate the importance of each input in this study (Fig 11). As observed, the fine aggregate (X_4_) is an important feature that positively impacts concrete compressive strength containing GGBFS. Precisely, with higher fine aggregate content, the compressive strength of concrete is improved. The fine aggregate and the cement contents are the most important input variables influencing the prediction accuracy of the ANN-SCG model. Regarding the effect, the difference between the fine aggregate and cement contents is relatively slight. In fact, the effect range by cement content is greater than that of fine aggregate content and sharply higher than that of the GGBFS content. The collected database can explain this observation, where the cement content, fine and coarse aggregate contents are the main constitution of concrete and the most gain of concrete compressive strength. The GGBFS is only a partial replacement of cement. Therefore, the gain of concrete compressive strength by GGBFS is less than that by the aggregates. Moreover, previous investigations of Tumidajski and Gong [62], or in Tsiskreli and Dzhavakhidze [63] show that the effect of fine aggregate on compressive strength is higher than that of the coarse aggregate. Therefore, the fine aggregate content has stronger effect on compressive strength than GGBFS content. The lowest impact on the prediction accuracy of ANN model is the Carboxylic-type hyperplasticizing content (X_6_), which also has a negative impact on the compressive strength. The lower the content of Carboxylic-type hyperplasticizing, the higher the compressive strength of concrete. The effect of superplasticizer content (X_7_) on the compressive strength of concrete is similar. These observations are in good agreement with the experimental investigation of Mazloom et al. [64]. At last, the water content (X_2_) has a negative impact, which means that the compressive strength of concrete decreases with higher water content. That is also confirmed in numerous investigations such as Oner and Akyuz [4], Shen and Xu [65], Zhou et al. [66]. Overall, the feature importance analyses are performed under the evaluation of the proposed ANN model. The feature importance analysis successfully simulates the effects of each input variable on the compressive strength of concrete containing GGBFS. Nonetheless, other types of sensitivity analysis such Partial Dependence Plot (PDP) [67] and Individual Conditional Expectation Plots (ICE) [68] need to be carried out to quantify and verify the effect of cement content, fine and coarse aggregate contents, and GGBFS content on the compressive strength value.

## 6. Conclusion

The goal of this study is to create a simple and effective ANN-SCG model for predicting the compressive strength of concrete containing GGBFS. To accomplish this goal, the ideal ANN design is first investigated, with two scenarios of hidden layer numbers ranging from 1 to 2. In each scenario, the number of neurons in each hidden layer is increased from one to fifteen. In total, 240 ANN structure alternatives are explored. On the basis of 595 examples gathered from the literature, 70% are chosen at random and utilized for training, while the remaining 30% are used for testing. For each scenario of ANN architecture, 500 simulations are performed. General statistical measures such as the coefficient of determination (R^2^), Root Mean Square Error (RMSE), and Mean Absolute Error (MAE) are used to evaluate the performance of each ANN architecture. The ANN architecture with 2 hidden layers, 14 neurons in the first hidden layer and 4 neurons in the second hidden layer, was discovered to be the best architecture for predicting the compressive strength of concrete containing GGBFS (the R^2^, RMSE, and MAE values are 0.965, 3.803, and 2.880, respectively, for the testing part). The sensitivity analysis shows the impact of each input variable on the output of the ANN-SCG model. The most important input variables are fine aggregate, cement content, testing age, and water content, which have distinctive effects on the model’s accuracy and should not be neglected while predicting the compressive strength. The findings of this study can be used to develop a dependable soft computing tool for precisely and rapidly predicting compressive strength. However, the accuracy of compressive strength prediction could be further improved by testing different machine learning techniques or optimization algorithms such as evolutionary algorithms, tree models, or support vector machine. Besides, quantifying the effects of the concrete constituents on the compressive strength should also be cross-checked with different sensitivity analysis techniques, namely the Partial Dependence Plot (PDP).

## Supporting information

S1 File (CSV)
